# Application and new findings of scRNA-seq and ST-seq in prostate cancer

**DOI:** 10.1186/s13619-024-00206-w

**Published:** 2024-10-29

**Authors:** Zhuang Li, Zhengnan Li, Yuanyuan Luo, Weiming Chen, Yinyi Fang, Yuliang Xiong, Qinyi Zhang, Dongbo Yuan, Bo Yan, Jianguo Zhu

**Affiliations:** 1 Department of Urology, Affiliated Hospital of Guizhou Medical University, Guiyang city, 550004 Guizhou Province China; 2 Department of Urology, Guizhou Provincial People’s Hospital, Guiyang city, 550002 Guizhou Province China; 3 Graduate School of Zunyi Medical University, Zunyi City, 563099 Guizhou Province China; 4 Medical College of Guizhou University, Guiyang city, 550025 Guizhou Province China

**Keywords:** Prostate cancer, Single-cell RNA sequencing, Spatial transcriptome sequencing, Tumor microenvironment, Heterogeneity, Treatment

## Abstract

Prostate cancer is a malignant tumor of the male urological system with the highest incidence rate in the world, which seriously threatens the life and health of middle-aged and elderly men. The progression of prostate cancer involves the interaction between tumor cells and tumor microenvironment. Understanding the mechanisms of prostate cancer pathogenesis and disease progression is important to guide diagnosis and therapy. The emergence of single-cell RNA sequencing (scRNA-seq) and spatial transcriptome sequencing (ST-seq) technologies has brought breakthroughs in the study of prostate cancer. It makes up for the defects of traditional techniques such as fluorescence-activated cell sorting that are difficult to elucidate cell-specific gene expression. This review summarized the heterogeneity and functional changes of prostate cancer and tumor microenvironment revealed by scRNA-seq and ST-seq, aims to provide a reference for the optimal diagnosis and treatment of prostate cancer.

## Background

Prostate cancer (PCa) exhibits the highest age-standardized incidence rate (29.4%) and age-standardized mortality rate (7.3%) among genitourinary tumors in men globally, according to *Global Cancer Statistics 2022* (Bray et al. 2024). Postoperative recurrence and metastasis are still great challenges for PCa treatment even with remarkable achievements of androgen deprivation therapy (ADT) and surgical resection in early-stage PCa (Simon et al. 2022).

PCa commonly has inconspicuous early symptoms and a poor prognosis in advanced stages, and relying solely on the studies of tumor cells is not completely sufficient for clinical treatment. The functional changes and spatial distribution characteristics of cells in the tumor microenvironment (TME) have a significant role in PCa proliferation, invasion and metastasis (Ge et al. 2022a, b; Yu et al. 2023). The conventional techniques of fluorescence-activated cell sorting and bulk RNA sequencing face challenges in analyzing and characterizing the cellular heterogeneity of PCa. Single-cell RNA sequencing (scRNA-seq) technology can capture the RNA expression profiles of individual cells, allowing for the construction of detailed cellular transcriptional profiles. Combined with the Kyoto Encyclopedia of Genes and Genomes (KEGG) pathway analysis, Gene Ontology (GO) analysis and cell trajectory analysis, scRNA-seq provided new insights into cell function, predicted signaling pathways, and elucidate cell differentiation or development processes (Khozyainova et al. 2023; Zhang et al. 2021). However, the spatial character of the original tissue was lost when cells were isolated. Spatial transcriptome sequencing (ST-seq) overcomes this limitation by capturing the RNA information of the cells for in situ sequencing (Zheng, Fang 2022). Applying scRNA-seq and ST-seq technologies in PCa research provides insights into the molecular mechanisms of tumorigenesis, invasion, metastasis, drug resistance and immune escape (Jin et al. 2021). The present review summarizes and discusses the potential value of these technologies in addressing PCa drug resistance, molecular targeted therapy and immunotherapy from current reports based on scRNA-seq and ST-seq in PCa and TME.

**Fig. 1:**
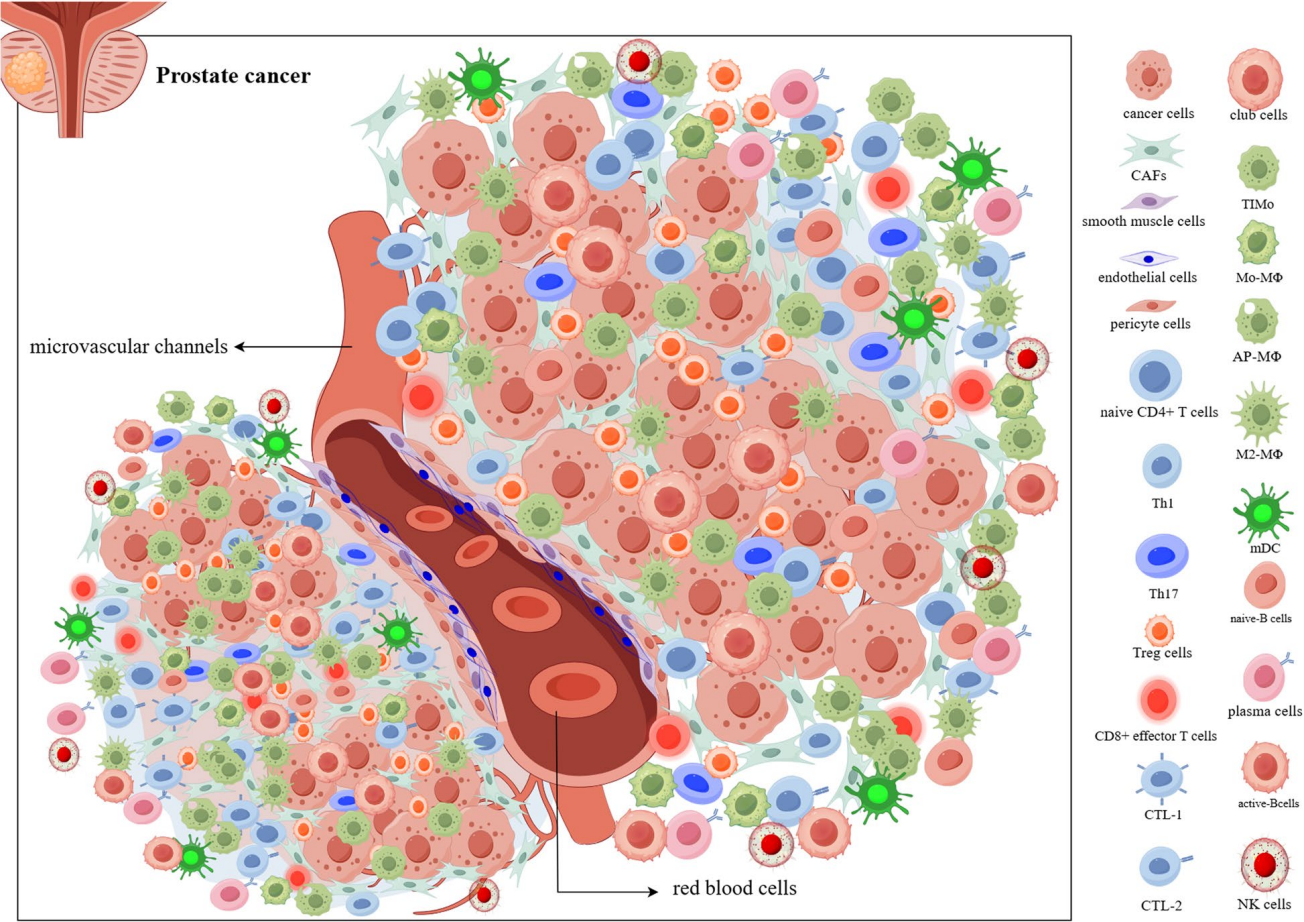
Cellular landscape of prostate cancer

As is shown in Figure 1, prostate cancer tissues mainly contain cancer cells, stromal cells and immune cells. Stromal cells include CAFs, smooth muscle cells, endothelial cells and pericytes. Immune cells included myeloid cells (CD16hi-Mo, TIMo, Mo-MΦ, AP-MΦ, M2-MΦ, mDC), CD4 + T cells (naive cells, T helper 1 cells, T helper 17 cells, regulatory T cells), CD8 + T cells (CTL-1, CTL-2 and CD8 +T cells), NK cells ( NKT, CD56 ^DIM^ NK, CD56^bright^ NK) and B cells (naive B, active B and plasma cells). CAFs are enriched in the marginal region of tumor cells. Vascular smooth muscle cells, pericyte cells and endothelial cells are implicated in the constitution of microvascular and lymphatic vessels in PCa. The TIME in PCa is characterized by an abundance of immunosuppressive myeloid cells, a decrease in T cells and activation of immunosuppressive Treg, an abundance of depleted CD56^DIM^ NK cells and B cell activation. The figure was generated by figdraw.com.

### ScRNA-seq reveals the landscape and function of normal prostate cells

Prostate cells are defined primarily based on cell shape and surface antigens, and comprehensive characterization of prostate cell types is lacking in conventional studies (Pletcher, Shibata 2022). ScRNA-seq technology provided a comprehensive view of global gene expression at the single-cell level, enabling a deeper investigation into the cell types and functions within PCa (Chen et al. 2022a, b; Vellky et al. 2024), which established a crucial foundation for further research in the field.

#### Epithelial cells

Epithelial cells of the prostate mainly include luminal cells and basal cells. Luminal epithelial cells (about 60 % of the epithelial cells) produce and secrete prostatic fluid to support male fertility (Crowley, Shen 2022; Ittmann 2018). Chen et al. (Chen et al. 2021) identified two luminal epithelial cells (*KLK3, KLK2*, *KRT8, KRT18*), one specific epithelial cell (*KRT8, KRT18*) and three unknown epithelial cells (*KRT13, KRT19*, *KRT15*, *KRT17*) using scRNA-seq technology. They combined immunofluorescence and gene detection to analyze the specific epithelial cells overexpressing *KRT8* and *KRT18*. Guo et al. (Guo et al. 2020) analyzed 8,545 cells from mouse prostate using scRNA-seq, luminal cells were classified into luminal-A (*Hoxb13, Abo, Spink1*), luminal-B (*Nkx3.1, Mmp7, Fgl1, Pbsn*) and luminal-C (*Tacstd2, Psca, Ck4*) cells using marker genes. Cell trajectory analysis of luminal cells using Monocle2 revealed that Luminal-C differentiates into Luminal-A and Luminal-B to reveal the stem cell characteristics of Luminal-C cells. Luminal-C was also detected in the human prostate by scRNA-seq and immunofluorescence staining. Further analysis of the Luminal-C cells revealed that Dist-Luminal-C cells (expressing *Tacstd2*/*YFP*) rebuilt the distal prostate luminal lineage through self-renewal and differentiation.

Basal cells (expressing *p63*, *CK5*, *CK14*) are located below the basement membrane of the epithelial cell layer to support and nourish epithelial cells (Pitzen, Dehm 2023). Research has shown that *Trop2* + basal cells possess stem cell characteristics and can produce luminal cells through cell division and differentiation, thus maintaining the renewal and repair of luminal cells (Graham et al. 2023; Shen et al. 2021). Wang et al. (Wang et al. 2020) have discovered that *Zeb1*+ basal cells in the early stages of mouse prostate development also exhibit stem cell characteristics and have the ability to differentiate into basal cells, luminal cells and neuroendocrine cells by using scRNA-seq. It has also been found that AR can antagonize *Wnt* activity in mouse prostate basal cells by binding to β-catenin, thereby maintaining the totipotency of basal stem cells (Horton et al. 2023). These results indicated an important role of basal cells in the differentiation, growth and repair of prostate epithelial cells.

Enhanced research on prostate stem cells helps reveal new features of prostate development. A scRNA-seq analysis of 98,000 cells from five prostates identified two new epithelial cells, *KRT5*/*KRT14*/*KRT13* hillock cells distributed in the central zone around the prostatic urethra, collecting ducts and ejaculatory ducts. The other was *KRT5*/*KRT8*/*SCGB1A1* club cells distributed in the prostatic urethra and collecting ducts (Henry et al. 2018). Other scholars have also found that *KRT13*+ hillock cells have stem cell characteristics using scRNA-Seq analysis of prostate cells in monolayer and organoid culture conditions (McCray et al. 2019). These studies enriched the epithelial cell landscape of prostate cells, especially the characterization and distribution of hillock cells and club cells, results contributed to a deeper understanding of the biological functions and potential clinical value of prostate epithelial cells.

#### Stromal cells

Prostate stroma is abundant in stromal cells and blood vessels, with stromal cells performing an essential role in maintaining the structural and functional stability of the prostate (Sanches et al. 2021). Fibroblasts in the prostate are involved in maintaining the structural integrity of the tissue (Pederzoli et al. 2023). Henry et al. (Henry et al. 2018) analyzed 4,156 stromal cells using scRNA-Seq technology to reveal fibroblasts secreted fibroblast growth factors (FGFs) and prostaglandins (PGs) to regulate the survival and growth of epithelial cells, while myofibroblasts are involved in the repair and immune response (Kwon et al. 2019). *APOD*+ fibroblasts were encapsulated around epithelial structures and expressed the secreted WNT inhibitors SFRPs and DKK1, while *C7*+ interstitial fibroblasts were dispersed in the extracellular matrix and expressed the WNT signaling regulator RSPO3 and growth factor (Joseph et al. 2021).

Smooth muscle cells are rich in actin and myosin, which control gland contraction, relaxation and substance metabolism. Three smooth muscle cells (*DES/ACTG2* + prostate smooth muscle cells, *THY1* + pericytes, *BCAM/MCAM* + vascular smooth muscle cells) were identified by scRNA-Seq technology, these cells are involved in prostate tissue development, neurogenesis and angiogenesis (Joseph et al. 2021).

#### Immune cells

The normal prostate stroma contains phagocytes, T-cells, NK-cells and B-cells, while previous studies have lacked detailed characterization of immune cell subtypes. Tuong et al. (Tuong et al. 2021) used scRNA-Seq technology to reveal the immune landscape of the prostate. Their studies revealed mononuclear phagocytes (MNPs) include monocytes, conventional dendritic cells (cDC1, cDC2) and macrophages (MAC1, MAC2, MAC-MT), which are involved in the removal of dead cells and tissue fragments and help maintain the homeostasis of prostate tissue. T cells are classified as naive T cells, memory T cells, CD4+ and CD8+ T cells, which are involved in immune surveillance and attack pathogens, infections and abnormal cells in the prostate. B cells have memory and naive B cells as well as plasma cells, while NK cells are consistent with those found in organs such as the liver and spleen. These immune cells are responsible for maintaining tissue homeostasis, fighting off pathogen invasion and regulating local immune responses in the normal prostate.

### ScRNA-seq and ST-seq reveal prostate cancer progression and tumor microenvironment changes

#### Reveal epithelial cell heterogeneity and pathological characteristics in PCa

##### Epithelial cells heterogeneity in PCa

Prostate epithelial cells are thought to be the cells of origin of PCa (Crowley, Shen 2022; Ittmann 2018). Abnormal proliferation and invasion of cancer cells result in severe disruption of the structure and function of the prostate (Advani et al. 2023). Bolis et al. (Bolis et al. 2021) analyzed the differentiation trajectory of PCa using scRNA-seq with the finding that *PIK3CA*, *TP53*, *FOXA1*, *KMT2C*, and *PTEN* gene point mutations promote the accumulation of AR signaling, which leads to epithelial cells mutating into primary PCa.

Epithelial cell types characterization by scRNA-seq improves PCa pathology diagnosis and informs optimization of clinical therapy regimens. Three types of prostate epithelial cells (luminal epithelial cells, basal epithelial cells, and neuroendocrine cells) and two new types (hillock cells and club cells) have been identified in previous studies, which were characterized as malignant and non-malignant (Henry et al. 2018). Song et al. (Song et al. 2022) have reported tumor-associated epithelial cells, including malignant luminal epithelial cells (*KLK2*, *KLK3*, *ACPP*, *NKX3-1*), malignant basal epithelial cells (*KRT5*, *KRT15*, *KRT17*, and *TP63*), neuroendocrine cells (*CHGB*, *KRT4*, *LY6D*) and club cells (*LTF/NKX3-1*^high^, *SCGB3A1/LCN2*^low^). Club cells with high androgen sensitivity may play a supporting role in the overall androgen-responsive cell environment of PCa, thereby promoting the occurrence of PCa, the study has not found hillock cells and hypothesizes that hillock cells may be depleted during PCa progression (Song et al. 2022). The newly discovered club cells promote the malignant progression of PCa and provide a new direction for the early diagnosis and treatment of PCa.

##### Molecular subtypes of PCa

Tumor molecular subtyping guides clinicians in designing personalized therapy regimens that can significantly improve overall survival. Different from breast cancer molecular subtypes (luminal A, luminal B, HER2-enriched and triple-negative), PCa molecular subtype identification was lacking in previous studies (Tsang, Tse 2020). Han et al. (Han et al. 2021) identified four molecular subtypes of PCa by using bulk and single-cell RNA sequencing techniques, including luminal A (adipogenic/AR-active/PSA-high ), luminal S (secretory/PAP-high), AVPC-I (immune-infiltrative) and AVPC-M (*Myc*-active). The first two are classified as luminal epithelial carcinoma, while the AVPC-I and AVPC-M were associated with PCa invasiveness. Their study improved the molecular classification of PCa relevant to therapy and hypothesized that the AVPC-I and AVPC-M subtypes are resistant to androgen receptor signaling inhibitors (ARSIs) and have low prostate-specific antigen (PSA) levels. Luminal A and AVPC-M were resistant to docetaxel and had high PSA/PAP ratios. This provides an excellent molecular basis for accurate medical treatment of PCa, which promises to enable personalized therapeutic approaches.

Gleason scores (GS) are key indicators for assessing the invasiveness and prognosis of PCa. Quan et al. (Quan et al. 2023) discovered that differential genes such as *NANS, PABPC1L, PILRB, PPFIA2* and *SESN3* contributed to the biological dysfunction of prostate cancer associated with GS progression using ST-seq. They also observed that epithelial tissues with the same GS in different patients had similar chromosome copy number variation (CNV), suggesting the same monoclonal origin. Ma et al. (Ma et al. 2020) performed scRNA-seq analysis on 7,904 cells from PCa tissues revealed the protein expression level of *HPN* was significantly higher in PCa with GS score >6 than that with GS score = 6, and hypothesized that *HPN* might be a diagnostic marker for PCa staging. ScRNA-seq technology has uncovered new molecular subtypes of PCa and identified Gleason score-related genes (*HPN*), this allows for an accurate assessment of tumor aggressiveness and prognosis, providing a more solid basis for clinical decision-making.

##### Pathologic characteristics of PCa

Adenocarcinoma is one of the most common pathologic types of PCa, and past studies have pointed to *MYC* as a major driver of prostate adenocarcinoma occurrence and progression (Surintrspanont, Zhou 2022). Qiu et al. (Qiu et al. 2022) combined scRNA-seq and chromatin immunoprecipitation sequencing data analysis to reveal that *MYC* overexpression antagonizes the classical AR transcriptional program, thereby promoting prostate carcinogenesis and progression to metastatic castration-resistant prostate cancer (mCRPC). The study indicated that targeted inhibition of *MYC* may help restore the normal transcriptional program of AR in PCa and increase the sensitivity of PCa to AR-targeted therapies. Fortunately, scientists have developed *MYC* inhibitors which can inhibit tumor growth in mice and increase tumor immune cell infiltration (Han et al. 2019). The *MYC* inhibitors will achieve clinical application and probably provide a breakthrough in PCa therapy in the future. These results guide a strategy for developing ARSIs in combination with other therapeutic options for advanced PCa.

Basal cell carcinoma (BCC) is relatively rare and with a generally poor prognosis in PCa, treatment of the BBC can be challenging (Low et al. 2023; Taskovska et al. 2023). Fortunately, researchers explored the characteristics and functions of BCC benefit from scRNA-seq technology, which may bring a breakthrough in the therapy of BCC. Hiroto et al. (Hiroto et al. 2022) used scRNA-seq combined with experimental analyses to discover that stromal AR promotes the occurrence of prostate basal epithelial carcinoma through IGFBP3-IGF1/IGF1R and Wnt/β-catenin regulatory pathways. Suppose molecular therapeutic targets are designed for the regulatory pathways found in this study, developing molecular targets jointly targeting tumor stromal cells and epithelial cells is possible. Other researchers analyzed PCa epithelial cells by scRNA-seq and found that basal cells overexpressing *KRT5, KRT14, KRT19* and *TP63* were associated with antigen processing and delivery (Chen et al. 2021). Similarly, the scRNA-seq analysis revealed that *CASC5, NUTM1, PTPRC, KMT2C* and *TBX3* were driver genes for mutations in prostate basal epithelial cancer (Su et al. 2020). These studies provide potential targets and new therapeutic ideas for both diagnosis and therapy of BCC.

Neuroendocrine prostate cancer (NEPC) is characterized by rapid proliferation, aggressiveness and insensitivity to ADT, which has a controversial origination (Yamada and Beltran 2021b). Using scRNA-seq in combination with genetic variation analysis, Wang et al. (Wang et al. 2022) elucidate that *MYC* amplification and *RB1* deletion can induce PCa, and NEPC might originate from *KLK3*-negative and untreated adenocarcinoma cells. A study also found that prostate cancer luminal-neuroendocrine transdifferentiation originated from the luminal epithelial, which didn't exclude a basal cell origin (Dong et al. 2020). Another study employed ST-seq to analyze AR pathway-positive prostate adenocarcinoma (ARPC) tissues and found that AR marker genes were significantly overexpressed in the ARPC region. Trajectory analysis demonstrates that the genetic map of ARPC and new NEPC is not continuous, suggesting that new NEPC does not originate from ARPC (Watanabe et al. 2023). The adeno-to-neuroendocrine transition in PCa can result in resistance to targeted therapies, analysis of genetically engineered mouse PCa samples using single-cell multiplexed genomics reveals that *Foxa2* regulates this transition pattern and activates the KIT pathway to promote tumor growth in both mouse and human NEPC, which offers a potential strategy for the treatment of castration-resistant NEPC (Han et al. 2022). These findings provide a new perspective for understanding the origin and pathological characteristics of NEPC and help guide treatment strategies in the future.

#### ScRNA-seq and ST-seq reveal that changes in TME promote PCa progression

The TME includes stromal cells and immune cells, the changes of these cells affect the proliferation, invasion, metastasis and immune regulation of PCa (Kang et al. 2022). Analysis of PCa tissue by using scRNA-seq reveals stromal cells (smooth muscle cells, endothelial cells) (Chen et al. 2021), cancer-associated fibroblasts (CAFs) (Liu et al. 2023) and immune cells(T cells, B cells, NK cells, dendritic cells, macrophages ) (Feriz et al. 2023; Ge et al. 2022a, b; Siefert et al. 2021; Tuong et al. 2021), changes in these cells promote PCa Progression. Some researchers used ST-seq to supplement and verify the results of scRNA-seq and found there were immune and non-immune cells (including fibroblasts, pericytes, and endothelial cells) around epithelial cells, while the proportion of immune cells (mainly monocytes, T cells, and plasma cells) in PCa tissues was significantly reduced (Hirz et al. 2023). The significant changes of these cells in TME are closely related to the progression of PCa.

##### Endothelial cells, pericytes and CAFs remodel tumor matrix to promote PCa progression

TME contributes to the progression of PCa besides cancer cells themselves. Xiao et al. (Xiao, Liang 2023) analyzed the scRNA-seq data of PCa and found that *HDGFL3* is an endothelial cell-specific gene. To combine in vitro cell and animal experiments to determine that *HDGFL3* + endothelial cells stimulate tumor angiogenesis, thereby promoting the growth and invasion of PCa.

ScRNA-seq and ST-seq techniques combined with GO analysis revealed that endothelial cells and pericytes were enriched in the PCa tumor region, in which endothelial cell-1 overexpressing the *HEY1*/*IGFBP3*/*FBLN5* genes was involved in vascularization and angiogenesis to promote cancer cell migration and proliferation, while pericyte-1 and pericyte-2 were involved in connective tissue development (Hirz et al. 2023).

CAFs play a central role in the TME and can promote cancer progression and recurrence in breast cancer and PCa (Fan et al. 2024; Lavie et al. 2022). With the application of scRNA-seq technology, the function and heterogeneity of CAFs in PCa were deeply resolved. Pan et al. (Pan et al. 2023) performed scRNA-seq analysis of twenty-six hormone-sensitive prostate cancer (HSPC) and eight castration-resistant prostate cancer (CRPC) samples to identify the two most abundant CAFs including *ACTA2 + CAV1 +* CAFs and *FN1 + FAP* + CAFs. The former is associated with the development of the microvascular system, while *FN1* + *FAP* + CAFs express collagen molecules thereby participating in extracellular matrix (ECM) remodeling. They also found cellular communication between endothelial cells and CAFs through CellPhoneDB analysis, it is not clear the different signaling pathways or molecular mechanisms are involved in these two cellular interactions. To better characterize the characteristics of CAFs in prostate cancer, Liu et al. (Liu et al. 2023) analyzed the scRNA-seq data of sixteen PCa samples to identify three kinds of CAFs with unique genetic characteristics including myofibroblastic CAF (myCAF), inflammatory CAF (iCAF) and antigen-presenting CAF (apCAF). The myCAF was found to be associated with ECM, cell adhesion and smooth muscle contraction, while iCAF acted in complement activation and epithelial cell proliferation as well as migration. Recently, some scholars have confirmed that *HSD17B2* in iCAF regulates AR activation and integrin β-like 1 secretion to promote migration, invasion and castration resistance of PCa cells (Zhang et al. 2023a, b). To explore the related characteristics of apCAF in PCa, Wang et al. (Wang et al. 2024) identified apCAF from PCa stroma based on the *CTSK* and *MRC2* marker genes to present MHC II-mediated antigens in the TME and to have a close signaling link with epithelial cells and T cells. The antigen presentation and processing functions of apCAF are markedly diminished after ADT and remodel the immunosuppressive TME, thereby promoting tumor immune evasion. A comprehensive description of markers specific to CAFs in PCa is lacking in previous studies. Pan et al. (Pan et al. 2024) combined scRNA-seq and multiple techniques to elucidate the characteristic genes (*ACTA2*, *ADAMTS2*, *ASPN*, *CDH11*, *COL1A1*, *COL1A2*, *COL3A1*, *COL5A1*, *CTSK*, *POSTN*, *RAB31*, *SFRP2*, *SPARC*, *THBS2*, *VCAN*) of CAFs in PCa, also demonstrating that CAFs are associated with PCa progression, higher GS scores and CRPC. Some CAFs also have anti-cancer effects in tumors, but there are few studies on PCa (Biffi, Tuveson 2021).

##### Immunosuppressive tumor microenvironment in PCa

The tumor immune microenvironment (TIME) is constructed with tumor-related immune cells and immunomodulatory factors, which impacts the progression and prognosis of PCa (Lv et al. 2022; Solinas et al. 2017). “Cold tumor” refers to a tumor that lacks sufficient immune cell infiltration in tumor tissue, and PCa is also a “Cold tumor” (Ma et al. 2022). This is one of the contributors to the poorer results of immunotherapy in PCa therapy, but the exact mechanism is unknown (Meng et al. 2023; Wu et al. 2022). With the application of scRNA-seq technology, there are greater works devoted to the analysis of cellular subtypes and novel functions of immune cells in PCa, providing design references for the research and intervention of immunotherapeutic strategies in the TIME (Tuong et al. 2021). Researchers used scRNA-seq to unsupervised cluster twenty-eight immune cell types in TIME and found two immune-related types of PCa, including PCa-immunity high (PC-ImH) and PCa-immunity low (PC-ImL) (Chen et al. 2022a, b). PC-ImH shows stronger immune properties and better prognosis compared to PC-ImL, which also signals a possible breakthrough in therapy by turning the prostate from “cold” to “hot”!

Myeloid cells are a type of immune cell derived from bone marrow stem cells that influence the malignant progression of a variety of tumors including breast cancer and lymphoma, which are considered to represent one of the most relevant cell populations for immunotherapy (Cheng et al. 2021). A scRNA-seq combined with ST-seq research reveals the state of the PCa tumor immune microenvironment (Hirz et al. 2023). (i) Immunosuppressive myeloid cell enrichment. They found that myeloid cells include non-classical monocytes (CD16hi-Mo, overexpressing *CD16*), tumor-inflammatory monocytes (TIMo, *CCL20, CD14, CCL2, CCL5*), monocyte-macrophage (Mo-MΦ, *S100A9*^*low*^and *C1QA*^*high*^), antigen-presenting macrophages (AP-MΦ, *APOE, APOC1, CTSF*), M2-macrophages (M2-MΦ, *CD68 + CD163* +) and myeloid dendritic cells (mDCs). Identified TIMo and M2-MΦ as immunosuppressive myeloid cells that lead to tumor progression. (ii) T-cell exhaustion and immunosuppressive Treg activity. They identified four CD4 + T cells (naive T cells, T helper 1 cells, T helper 17 cells, regulatory T cells) and three CD8 + T cells (CTL-1, CTL-2, CD8 + T cells) from T cells. Cytotoxic T lymphocyte-1 (CTL-1) and CD8 + T cells showed higher T cell exhaustion genes. (iii) Exhausted CD56^DIM^ NK cells. CD56 ^DIM^ NK cell overexpression exhaustion genes (*FGFBP2, GNLY, GZMB, GZMH*) were identified from natural killer T cells (NKT), CD56 ^DIM^ NK cells, CD56^bright^ NK cells and CD56^bright^-IL7R + NK cells. (iv) Activated B-cells. The B cell activity of naive B, active B and plasma cells in cancer and adjacent tissues was significantly higher than normal prostate. They used ST-seq to confirm that PCa is abundant in B cells and macrophages, but poor in monocytes, T cells and NK cells. In conclusion, the article reveals the pattern of immunosuppressive TME in PCa and explains the “cold tumor” and “immune desert” characteristics.

Macrophages are myeloid cells that are immunologically related to tumors and are often divided into two opposing roles called M1 (tumor growth inhibiting) and M2 (tumor growth promoting) macrophages, with tumor-associated macrophages (TAMs) recruited in TME exhibit M2 properties (Guan et al. 2024). Ou et al. (Ou et al. 2024) analyzed scRNA-seq data and weighted correlation network analysis (WGCNA) to reveal that M2 macrophage-associated genes (*PLPP1*, *SMOC2*, *ABCG1*, *HES1*, *GPR160*, *MAZ*, *STMN1*, *EPCAM* and *MYC*), and that M2 macrophages contribute to the proliferation, invasion and migration of PCa. Another report analyzed an immune cell transcriptional landscape using scRNA-seq and found lipid TAMs secrete *CCL6* to drive tumor growth and invasiveness in PCa. (Masetti et al. 2022). Unlike this macrophage feature, Tuong et al. (Tuong et al. 2021) found a prostate-specific, zinc transporter-expressing macrophage population (MAC-MT) from PCa tissues, which can reduce the accumulation of zinc ions in cells. The MAC-MT secretes T cells to recruit chemokines to exert anti-tumor effects. Salachan et al. (Salachan et al. 2023) used ST-seq to find that helper, cytotoxic and regulatory T cells (expressing *CD4, CD8*, *FOXP3*) are surrounded by the immunosuppressive TME, which prevented the toxic effects of T cells on tumor cells. This study confirmed that CAFs surround epithelial cells (expressing *KLK2*, *KLK3*, *KLK4*) and prevent efficient migration of immune cells to malignant epithelial cells. Similarly, Feriz et al. (Feriz et al. 2023) found that the number of tumor-infiltrating dendritic cells (TIDC) in PCa tissues was higher than the normal tissues. TIDC can produce chemokines (CCL2 and CCL3), cytokines (TNF-α and IL-6) and growth factors (VEGF and CCL5), which promote tumor angiogenesis and cancer cell proliferation as well as metastasis. In summary, the scRNA-seq and ST-seq analyses contribute to a better understanding of the heterogeneity in immune cells and immunosuppressive TME characteristics in prostate cancer TIME, which facilitates the development of new strategies for PCa immunotherapy.

#### The potential mechanism of PCa distant metastasis

PCa faces difficulties in therapy and poor prognosis after the occurrence of invasive or distant metastases (including bones, lymph nodes, lungs and liver) (Kang et al. 2022). Fortunately, the development of targeted therapies (radium-223 chloride, zoledronic acid) for the treatment of PCa bone metastases has been achieved, remains a need to continue the search for therapeutic strategies that can significantly inhibit distant metastasis of PCa (He et al. 2022).

The distant metastasis of PCa is a complex process involving multiple cellular and molecular mechanisms. Xin et al. (Xin et al. 2023) performed the scRNA-seq analysis of tissues from PCa primary lesions and lymphatic metastases to reveal the heterogeneity of luminal cells, tumor-infiltrating immune cells and fibroblasts contributed to the specific TME of PCa metastases. The *MYC* acted as an oncogene and was enriched in tubulointerstitial cells to promote PCa metastasis by inducing an immunosuppressive TME through PD-L1 or CD47. CD8 + T cells perform antitumor roles in tumor immunity. However, T cell depletion in PCa and *CCR7*+/*IL7R*+CD8+ T cells exert immunosuppressive roles in promoting PCa progression. At the same time, *STEAP4+/ADGRF5*+ fibroblasts and *CXCR4+/SRGN*+ fibroblasts alter tumor cells and immune cells in TME to promote lymphatic metastasis in PCa. It is possible to design targeted therapies against specific cells in the TME (such as *STEAP4*+ *ADGRF5*+ fibroblasts) to block or slow down the metastatic process of PCa and to improve the therapeutic efficacy and survival of patients.

The difficulty of research on bone metastasis of PCa is the lack of tumor cell characteristics and corresponding tumor microenvironment models. Arriaga et al. (Arriaga et al. 2020) developed an *NPK*^EYFP^ (*Nkx3.1*, *Pten*, *Kras*) mice to overcome this limitation. They performed scRNA-seq analysis on PCa and metastasis tissues (bone, lymph node, lung, liver and brain) of *NPK*^EYFP^ mice to determine that the co-activation of *MYC*/*RAS* pathways is a key driver of bone metastasis of PCa. This work provided a key model for further research of distant metastasis in PCa and new ideas for targeting *MYC* and *RAS* pathway therapy strategies. Another study also indicated that *CRISP3* is a key gene associated with desmin expression in bone metastatic prostate cancer (BMPCa), which promotes PCa proliferation and metastasis through the advancement of epithelial-mesenchymal transition (EMT), and that *CRISP3* could serve as a therapeutic target to inhibit PCa progression (Zhang et al. 2023a, b).

The clinical practice guides therapy and evaluates the survival of patients with metastatic PCa mainly based on PSA level, pathologic stage and GS score (Cao et al. 2022; Jeong et al. 2021). To improve the treatment of metastatic PCa, Thysell et al. (Thysell et al. 2022) divided metastatic PCa into three subtypes, including MetA, MetB and MetC according to the transcriptome characteristics of metastatic PCa. The most common MetA showed high AR activity and a relatively good prognosis after treatment, while MetB and MetC showed low AR activity. MetB has higher cell cycle activity with the presence of untreated CRPC and liver metastases, suggesting that MetB patients tend to have the worst prognosis. This finding provides clinicians with options for more targeted therapy with MetA potentially being more suitable for ADT, while MetB may require a combination of chemotherapy and targeted therapy.

#### Epigenetic characterization of prostate cancer revealed

PCa also has a genetic characterization, the *HOXB13*, *BRCA2*, *CHECK2*, *ATM*, *MMR*, *BRCA1*, *PALB2*, *BRP1* and *NBS1* gene mutations are considered to be common markers of hereditary prostate cancer (HPCa) (Tian et al. 2021; Vietri et al. 2021). Epigenetic inheritance involving altered DNA methylation and histone modifications has been strongly associated with PCa development and progression in recent years (Kumaraswamy et al. 2021). Interleukin-1 receptor-associated kinase 1 (IRAK1) is a key signaling molecule in the Toll-like receptor (TLR) pathway (Schagdarsurengin et al. 2022). Zhang et al. (Schagdarsurengin et al. 2022)found that IRAK1 is enriched in luminal epithelial cells and is regulated by the epigenetic state of the IRAK1 differentially methylated region (IRAK1-DMR) to exert an anti-apoptotic effect in luminal epithelial cells by using the analysis of scRNA-seq data in PCa. Designing agonists against IRAK1 targets optimizes therapy strategies for prostate adenocarcinoma. The NEPC has an elusive molecular characteristic and faces difficulties in correct diagnosis. Wang et al. (Wang et al. 2022) analyzed scRNA-seq data from HSPC and CRPC, revealing that epigenetic reprogramming drives the differentiation of androgen-dependent prostate cancer (ADPC) into NEPC at different stages and that genes such as *ASCL1*, *FOXA2*, *NKX2-2*, *POU3F2* and *SOX2* play key roles in the differentiation process.

### Implications of scRNA-seq and ST-seq for optimizing PCa treatment regimens

Radical prostatectomy is the preferred option for localized PCa, but local recurrence or distant metastasis may also occur after surgery. Advanced and metastatic PCa results in therapy with ADT, ARSIs (enzalutamide) and chemotherapy (docetaxel), but resistance or progression eventually (He et al. 2022; Sekhoacha et al. 2022). Molecularly targeted therapies and immunotherapies that attack specific targets in the tumor or enhance the immune system to eliminate tumor cells are promising breakthroughs in PCa (Grüllich et al. 2020).

#### Reveal the objective drug resistance mechanism of PCa

ARSIs are commonly used in the treatment of advanced PCa, but most PCa patients will eventually develop resistance to these drugs (Aurilio et al. 2020). The most common mechanism of PCa or CRPC resistance to ARSIs and ADT is typically associated with overexpression or mutation of genes of the AR and its pathways.

Fortunately, researchers have provided more objective mechanisms of drug resistance with the help of scRNA-seq and ST-seq techniques. Taavitsainen et al. (Taavitsainen et al. 2021) performed scATAC-seq analysis on prostate parent cells (LNCaP or LNCaP-ENZ48) and drug-resistant cells (RES-A or RES-B) to reveal chromatin reprogramming is the basis of enzalutamide resistance, and this resistance is related to the reconstruction of transcription factor DNA motifs. They identified pre-existing and persistent cells associated with PCa relapse that existed before resistance to ARSIs therapy developed using scRNA-seq combined with ST-seq technology to find them were enriched in PCa tissue. It is hypothesized that these two types of cells with regenerative properties and persistence reduce responsiveness to ARSIs therapy to promote resistance. Fan et al. (Fan et al. 2023) used the same technique to find that *ZNF337, MAPK15* and *ESRRG* genes suggest a good prognosis in the early stage of enzalutamide treatment, while *CCDC150, CCDC18* and *POC1A* have a poor prognosis. It also indirectly indicates the pre-existing drug resistance during the treatment of PCa. An analysis of ScRNA-seq carried out on primary PCa and CRPC/mCRPC samples also revealed that pre-existing neuroendocrine and CRPC-like cells in PCa may be resistant to ARSIs, which could promote the progression of prostate cancer to CRPC, another study also pointed out that neuroendocrine cells with low AR expression have higher proliferation and invasion potential in PCa (Cheng et al. 2022; Su et al. 2022).

Taxanes inhibit cell division by interfering with the normal function of microtubules, thereby preventing the proliferation of cancer cells (Mazumder et al. 2022). Docetaxel is one of the preferred chemotherapy drugs for CRPC treatment, but the probability of drug resistance is not uncommon. To better understand the mechanism behind the acquisition of drug resistance. Schnepp et al. (Schnepp et al. 2020) performed scRNA-seq on docetaxel-sensitive and docetaxel-resistant mutants of DU145 and PC3 prostate cancer cell lines to determine that nuclear protein 1 (NUPR1) is a driver of docetaxel resistance. Several effective combination drug therapies have also been identified using scRNA-seq technology, including topotecan combined with docetaxel (Mitra Ghosh et al. 2023), trichostatin-A combined with docetaxel (Schnepp et al. 2021) and shikonin combined with docetaxel (Fan et al. 2023). Unfortunately, these combinations have failed to reveal specific mechanisms for alleviating or overcoming drug resistance, and extremely effective combination drug regimens have not been found. Therefore, it is necessary to intensify research in the field to identify safe and effective drug regimens to address the problem of drug resistance.

#### Developing new molecular therapeutic targets

The CAFs can regulate tumor growth, metastasis, therapeutic response and drug resistance in the TME of PCa (Bonollo et al. 2020). The studies of the cellular signaling communication and regulatory mechanisms of CAFs in PCa and TME using ScRNA-seq technology reveal many molecular targets for the therapy of PCa (Wang et al. 2023; Xu et al. 2024; Zhang et al. 2023a, b). In the study published in *Cancer Cell*, Wang et al. (Wang et al. 2023) found that ADT-induced *SPP1* myofibroblasts originated from iCAF in HSPC. ADT treatment releases a TGF-β signal, which leads to *SOX4-SWI/SNF-*dependent CAFs phenotype conversion, and *SPP1* myofibroblasts make PCa fail to ADT through the *SPP1-ERK* paracrine mechanism. Therefore, targeting this *SPP1* myofibroblast inhibits CRPC, and this new therapeutic target has the opportunity to solve the problem of PCa resistance. Zhang et al. (Zhang et al. 2023a, b) also found that *HSD17B2* in CRPC can regulate CAFs function and promote PCa metastasis through the *AR/ITGBL1* axis. This work also nicely indicates that *HSD17B2* in CAFs may be a promising target for the therapy of CRPC. In another study, Xu et al. (Xu et al. 2024) integrated scRNA-seq and bulk RNA sequencing to establish a prognostic model of CAFs-related miRNAs (such as hsa-miR-106b-5p, hsa-miR-222-3p, hsa-miR-493-5p) and revealed that the down-regulation of hsa-miR-106b-5p could suppress the proliferation and migration of PCa cells. The prognostic model of CAFs-associated miRNAs provides new insights into PCa individualized and precision therapy, which enriches the potential therapeutic targets for PCa.

With the help of scRNA-seq technology, it was also found that tumor stromal cells contributed significantly to prostate cancer metastasis and ADT treatment resistance. Heidegger et al. (Heidegger et al. 2022) performed scRNA-seq analysis on four cases of PCa tissues to find endothelial cells that promote the formation of arteries, veins and immature blood vessels. They combined cell experiments and found that *CXCR4* antagonist AMD3100 can effectively inhibit the proliferation and migration of tumor endothelial cells. Wen et al. (Wen et al. 2023) analyzed the scRNA-seq data of PCa and found that *HOPX, LY6E, THBS1, LDHA, CRISPLD2, FOXC1, SAMD4A, NR4A1, MYC* and *UBE2S* were all involved in the occurrence of PCa. In addition, studies using scRNA-seq technology have found many promising new targets for the treatment of PCa, including *HER3* (Gil et al. 2021), *ORC1* (Li et al. 2024), *HSPH1* (Wu et al. 2024), *MAD2L1* (Wang et al. 2022) and *MXRA8* (Miao et al. 2024). The ability to study these potential therapeutic targets deeply may bring hope for PCa therapy by discovering specific molecular targets for PCa treatment in the future.

#### Optimized immunotherapy regimen

PCa lacks sufficient immune cell infiltration, there are multiple mechanisms for tumor cells to evade immune system attack (Angappulige et al. 2024). With advances in immunotherapy technology and an increased understanding of the PCa immune microenvironment, it is possible to explore many potential immunotherapies for PCa.

Immune checkpoint inhibitors (ICIs) are a type of molecules that block immune checkpoints (ICs) to relieve the inhibition of the immune system on cancer cells, thereby enhancing the ability of T cells to attack cancer cells (Naimi et al. 2022). The classical immune checkpoints of PD-1 and PD-L1 of ICIs have achieved significant efficacy in treating many cancers, but most PCa patients are not sensitive to the therapy (Xu et al. 2021; Yi et al. 2022). Hawley et al. (Hawley et al. 2023) performed scRNA-seq analysis on the metastasis sites of mCSPC patients treated with ADT and anti-PD-1 to found that *TNF +* CD4-Tregs, *LAG3 +* CD8 + T cells and *GITR* + Tregs were significantly increased in mCSPC tissues and metastasis tissues (bone, lymph node and liver) after treatment, which indicated that ICIs combined with ADT could change the pattern of TIME in PCa. The CAFs are responsible for the communication between tumor cells, immune cells and stromal cells in tumor tissues (Saw et al. 2022). A single-cell and bulk RNA sequencing study found that CAFs up-regulate the overexpression of immune checkpoint molecules (such as *PD-1, PD-L1, CTLA4/B7* ) in high-risk PCa cells in TME, thereby inducing CD8 + T cells dysfunction and assisting tumor cells in immune escape (Liu et al. 2023). The combination of ICIs therapy with other drugs (chemotherapy, vaccines, ARSIs) for PCa become a new strategy for PCa therapy in the future.

T-cell infiltration in TME performs a double role in attacking tumor cells or promoting tumor cell growth, which depends on the immune status of the TME and the type of T-cells. Developing immune cell therapies for PCa could be better facilitated by an in-depth analysis of its mechanisms, considering its “immune-desert” character. Feriz et al. (Feriz et al. 2023) used scRNA-seq analysis to find that *CCR5/CCL5, CD52/SIGLEC10* and *HLA-DPB1/TNFSF13B* were involved in the migration of immature dendritic cells (DCs) to the TME and disrupted the antigen-presenting function of DCs, inducing effector T-cell dysfunction. Peng et al. (Peng et al. 2022) successfully identified *EP4* as a specific target for PCa immunotherapy by scRNA-seq technology to prove that the novel *EP4* antagonist (YY001) enhances the proliferation and anti-cancer function of T cells, inhibiting differentiation and maturation of myeloid cells enhances synergistic effects of anti-PD-1 antibodies. The deepening of research on the molecular characterization of PCa and TME is expected to identify factors that impede PCa immune infiltration to provide a more effective therapy for immunotherapy.

#### Revealing the role and therapy potential of prostate cancer stem cells

Prostate cancer stem cells (PCSCs) have unique self-renewal and multidirectional differentiation properties, which are considered to drive prostate cancer therapy resistance and tumor recurrence (Verma et al. 2023). Previous research has found that PCSCs may originate from basal or luminal progenitor/stem cells, which differentiate into tumor cells with different phenotypes and functions in TME (Gupta et al. 2019; Zhang et al. 2016; Zhang et al. 2018). A comprehensive summary of the identification of PCSCs by techniques such as scRNA-seq, flow cytometry, genomics and epigenetics is presented by Su et al. (Su et al. 2024). These PCSCs include basal stem cells (*CD49f/CD133/Bcl-2*, *KRT16/17/6*, *CK5/CK14/p63*, *hTERT*, *Trop2*, *CD117*) and luminal stem cells (*CK8/CK19/CK18/CD26*, *Tacstd2/CK4/PSCA*, *NKX3.1*, *EZH2*, *ABCG2*) (Su et al. 2024). PCSCs are formed by prostate progenitor/stem cell gene mutations or changes in the TME and exhibit self-renewal and metastatic capabilities (Gupta et al. 2019). The expression of AR by PCSCs is absent or reduced, leading to the emergence of resistant PCa and CRPC (Di Zazzo et al. 2016; Verma et al. 2023).

The therapies including ADT and chemotherapy for PCa are aimed at eliminating large numbers of conventional tumor cells, but this is probably ineffective for resistant PCa and CRPC. Cancer stem cell therapy has also received plenty of attention in recent years, and it has the potential to provide a breakthrough in prostate cancer therapies (Escudero-Lourdes et al. 2022). An integrated scRNA-seq, ST-seq and bulk ATAC-sequence analysis reveals *SOX9*^*High*^*AR*^*low*^ club cells exhibiting stem cell properties after ADT therapy, which redifferentiating into drug-resistant and invasive CRPCs (Bian et al. 2024). Several studies have found that aberrant expression of *SOX9* is involved in PCa development and progression, drives the transformation of club cells into stem cells, and that inhibition of *SOX9* expression improves the functional characteristics of drug resistance and aggressiveness in PCa (Bian et al. 2024; Nouri et al. 2020). Another study integrating ATAC-seq, RNA-seq, and DNA sequencing in CRPC revealed four CRPC subtypes, including CRPC-AR (rich in AR features), CRPC-WNT (rich in Wnt signaling), CRPC-NE (rich in NE features) and CRPC-SCL (rich in stem cell features) (Tang et al. 2022). The YAP, TAZ, TEAD and AP-1 proteins interact with each other and drive the oncogenic growth of CRPC-SCL, and inhibitors of the YAP/TAZ pathway (Vitexoporfin) and c-Fos/AP-1 inhibitor (T-5224) may be effective in inhibiting the proliferation, invasion and metastasis of CRPC-SCL (Tang et al. 2022). However, cancer stem cell therapies are not 100% effective due to limitations imposed by the small number of cancer stem cells and the heterogeneity of cancer stem cells, which requires a combination of other therapies to benefit a larger number of patients.

### Conclusions and perspectives

The abnormal proliferation and differentiation of cancer cells are the core of the formation and development of PCa. The scRNA-seq and ST-seq were used to analyze the heterogeneity of PCa and TME, identify the cell types and their marker genes in PCa tissues, and reveal the location and function of these cells in the TME (Table 1). These greatly promote the diagnosis and treatment of PCa in the future.

**Table 1:** ScRNA-seq and ST-seq reveal the cell types and markers, location/characteristics and function in prostate cancer

Cell types			Markers	Location/ characteristics	Functions
Cancer cells	Luminal epithelial cells		KLK2, KLK3, ACPP, NKX3-1	Acinar and glandular ducts	Upregulated AR signaling, mutant to prostate adenocarcinoma
	Basal cells		KRT5, KRT15, KRT17, TP63	Close to the luminal epithelial cells	Stem cell characteristics, rare origin of cancer cells
	Neuroendocrine cells		CHGB, KRT4, LY6D	Scattered distribution in tumor tissues	Promote tumor growth and invasion
	Club cells		PIGR, MMP7, CP, LTF	Central and transition zones	Primed for tumor cell transformation and may also promote prostate tumorigenesis
Stromal cells	ACTA2+ CAV1+ CAFs		ACTA2, CAV1, MYH1, MCAM, RGS5	Extracellular matrix	Related to microvascular system development
	*FN1*+ *FAP*+ CAFs		FN1, FAP, DCN, COL1A1, COL3A1		ECM remodeling
	myCAF		ACTA2, TAGLN, MYL9, COL1A2, FAP		ECM remodeling, collagen metabolic process, cell adhesion, and smooth muscle contraction
	iCAF		CXCL12, IGF1, C3, C7, CFD, CFH		Complement activation, promotes epithelial cell proliferation and migration
	apCAF		CD74, HLA-DRB1, HLADRA		Antigen processing and presentation
	Endothelial cells		HDGFL3, HEY1, IGFBP3, FBLN5		Involved in vascular development and angiogenesis
	Pericyte cells		NG2, PDGFRβ, ACTA2		Involved in extracellular structural organization and connective tissue development
	Smooth muscle cells		ACTA2, MYH11, RGS5		Structural support and blood flow regulation
Immune cells	Myeloid cells	CD16hi-Mo	CD16^high^	Immunosuppressive myeloid cells are enriched in prostate cancer	Not yet fully defined
		TIMo	CCL20, CD14, CCL2, CCL5		Secretion of pro-inflammatory cytokines and immunosuppressive
		Mo-MΦ	S100A9^low^, C1QA^high^		Transformation of monocytes to macrophages
		AP-MΦ	APOE, APOC1, CTSF		Antigen processing and presentation
		M2-MΦ	ARG1/2, CD32, CD163, CD23*,* CD200R1*,* EGFL7, LYVE1, NRP1		High infiltration of M2 macrophages is associated with tumor recurrence and metastasis
		mDC	CD1C, CLEC9A, LAMP3		Presenting tumor antigens to T cells
	CD4+ T cells	naive CD4+ T cells	CD4, CD45	Low T-cell infiltration in PCa, T-cell depletion, and immunosuppression Treg activation	It can differentiate into Th1, Th17 or Treg.
		Th1 cells	TBX21, IFN		Activate macrophages and promote cellular immune response
		Th17 cells	*RORC*, IL-17A		Promote inflammatory response and neutrophil recruitment
		Treg cells	FOXP3, TNFRSF9, TNFRSF18, TNFRSF4		Secreting inhibitory cytokines and regulating immunosuppression
	CD8+ T cells	CTL-1	CD8, CD27, CD28		Release cytotoxic molecules to destroy the cell membrane of tumor cells
		CTL-2	CD8, CD45RO, CD62L		Fulfillment of the role of memory T cells to enhance the durability of the immune response.
		CD8+ effector T cells	CD8, CD45RA, CD69		Identify and attack tumor cells
	NK cells	NKT cells	CD3D^high^, CD8^high^, CD56^Dim^	Enriched in exhausted CD56^Dim^ NK cells	Recognizes lipid antigens and kills tumor cells by Releasing cytokines and cytotoxic molecules
		CD56^Dim^ NK cells	HAVCR2^high^		Potent cytotoxicity
		CD56^bright^ NK cells	XCL1, XCL2, GZMK, CD44, KLRC1		Involved in the regulation of immune and inflammatory responses
		CD56^bright^-IL7R + NK cells	IL7R, CD62L		Promotes NK cell survival and function
	B cells	naive-B cells	CD19, CD20, CD22	B cell activation in the TME	Differentiate into active B cells
		active-B cells	CD27, CD80, CD86		Differentiate into plasma cells; act as antigen-presenting cells, activate T cells
		plasma cells	CD138, CD38		Killing of tumor cells through antibody-dependent cell-mediated cytotoxicity effects

The scRNA-seq technology has revealed different molecular subtypes such as Luminal A, Luminal S, AVPC-I and AVPC-M in PCa (Han et al. 2021). It has also revealed two immune-related subtypes of PCa (PC-ImH and PC-ImL) (Chen et al. 2022a, b), and three subtypes of MetA, MetB and MetC in metastatic PCa (Thysell et al. 2022). According to the characteristics of these molecular subtypes of PCa, Luminal A and Luminal S are sensitive to androgen, and the use of ADT combined with docetaxel or ADT combined with ARSIs to help prevent PCa metastasis and improve prognosis (Mandel et al. 2023; Wala et al. 2023). Treatment of immune-related AVPC-I, AVPC-M, PC-ImH and PC-ImL, using ICIs combined with ARSIs, tyrosine kinase inhibitors, radium-223, radiotherapy and tumor vaccines to overcome the immune resistance of PCa and improve the clinical outcome as well as overall survival of patients (Kgatle et al. 2021; Li et al. 2023; Mulvey et al. 2022). The key to MetA, MetB and MetC therapy is to diagnose and treat them early, and patients might benefit from metronomic chemotherapy in combination with ICIs and radium-223 (Lowrance et al. 2023; Wysocki et al. 2022; Yamada and Beltran 2021a).

Cancer stem cells with self-renewal and multilineage differentiation as drivers of PCa tumorigenesis, recurrence, metastasis and prognosis (Wolf et al. 2022). The scRNA-seq revealed stem cell properties of *TROP2* + basal cells and club cells in PCa tissues, which continuously renew and differentiate into luminal cells, basal cells and neuroendocrine cells (Koukourakis et al. 2023). Therapeutic strategies with stem cells to inhibit AR and its associated signaling pathways, thereby addressing the issue of drug resistance in prostate therapy. Therapy that targets these stem cells would probably achieve unexpected therapeutic results.

CAFs are the most abundant stromal cells in PCa, ST-seq analysis found that CAFs are mainly distributed around tumor cells and may form a physical barrier to protect tumor cells from T cell attack (Yu et al. 2023). CAFs influence the proliferation, migration, and invasive ability of tumor cells through secreting cytokines, growth factors and extracellular matrix components, and also secreting immunosuppressive factors to inhibit the activity of T cells and promote tumor immune escape (Bedeschi et al. 2023). CAFs provide favorable conditions for tumor growth and metastasis through mechanisms that promote angiogenesis, ECM remodeling, and inflammatory responses. The complex functions of CAFs undoubtedly increase the difficulty of PCa treatment, but also provide new therapeutic ideas for molecular targeted therapy. The ability to achieve inhibition of the CAFs activity or apoptosis through the use of CAFs-associated therapeutic targets (*HSD17B2*, *HOPX*) or the ability to directly target inhibition of *ACTA2*+ *CAV1*+ CAFs, *FN1*+ *FAP*+ CAFs, myCAFs and iCAFs could lead to dramatic advances in PCa therapy.

Cancer immunotherapy is a therapy that relies on the human immune system to recognize and attack cancer cells, but the treatment is not effective in PCa (Sridaran et al. 2023). As a “cold tumor”, PCa is characterized by increased immunosuppressive myeloid cells, T cell depletion, immunosuppressive Treg activation, low infiltration of NK cells, B cell activation in the TME and lack of effective ICs (Hirz et al. 2023). Immunotherapies such as PD-1/PD-L1 and CTLA-4 inhibitors are currently in the experimental phase, and only sipuleucel-T has been approved for clinical application in the United States (Mitsogiannis et al. 2022). To better develop immunotherapy for PCa, insightful research on the immune mechanisms in the TME of PCa is needed to explore new immune checkpoints or signaling pathways to improve the efficacy of immunotherapy.

The scRNA-seq is a useful tool to explore the complexity of cellular heterogeneity, which still suffers from limitations such as cell loss or degradation caused by the cell capture process, difficulty in adequately identifying rare cells, and inability to provide information on the spatial location of cells (Kharchenko 2021). Therefore, there is a requirement to improve cell capture and processing techniques, enhance single-cell resolution and incorporate ST-seq techniques to obtain more comprehensive cellular information. Although ST-seq techniques allow gene expression information to be obtained at spatial resolution from the original tissue, they have disadvantages such as relatively low resolution and insufficient sequencing depth, which require technical innovations to overcome (Du et al. 2023; Feng et al. 2024). The combination of scRNA-seq and ST-seq with multi-omics technologies such as proteomics and metabolomics will help to understand the molecular mechanisms of PCa recurrence and metastasis more comprehensively, discover more molecular targets associated with resistance to PCa therapies to achieve more effective therapeutic regimens and better prognosis for patients (Baysoy et al. 2023).

## Data Availability

Not applicable.

## Abbreviations

PCa: Prostate cancer
scRNA-seq: Single-cell RNA sequencing
ST-seq: Spatial transcriptome sequencing
TME: Tumor microenvironment
KEGG: Kyoto Encyclopedia of Genes and Genomes
GO: Gene ontology
AR: Androgen receptors
EMT: Epithelial-mesenchymal transition
MNPs: Mononuclear phagocytes
GS: Gleason score
CNV: Copy number variation
BCC: Basal cell carcinoma
mCRPC: Metastatic castration-resistant prostate cancer
NEPC: Neuroendocrine prostate cancer
ARPC: Androgen receptor pathway-positive prostate adenocarcinoma
CRPC: Castration-resistant prostate cancer
BMPCa: Bone metastatic prostate cancer
HPCa: Hereditary prostate cancer
HSPC: Hormone-sensitive prostate cancer
ADPC: Androgen-dependent prostate cancer
CAFs: Cancer-associated fibroblasts
myCAF: Myofibroblastic CAF
iCAF: Inflammatory CAF
apCAF: Antigen-presenting CAF
ECM: Extracellular matrix
TIME: Tumor immune microenvironment
TAMs: Tumor-associated macrophages
WGCNA: Weighted correlation network analysis
IRAK1: Interleukin-1 receptor-associated kinase 1
TLR: Toll-like receptor
CTLs: Cytotoxic T lymphocytes
PC-ImH: PCa-immunity high
PC-ImL: PCa-immunity low
MAC-MT: Prostate-specific, zinc transporter-expressing macrophage population
TIDC: Tumor-infiltrating dendritic cells
ADT: Androgen deprivation therapy
ARSIs: Androgen receptor signaling inhibitors
AR-V7: AR splice variant 7
NUPR1: Nuclear protein 1
ICIs: Immune checkpoint inhibitors
ICs: Immune checkpoints
DC: Dendritic cells
PCSCs: Prostate cancer stem cells
AVPC-I: Aggressive variant prostate cancer-immune infiltrative
AVPC-M: Aggressive variant prostate cancer-Myc active
CD16hi-Mo: Non-classical monocytes
TIMo: Tumor-inflammatory monocytes
Mo-MΦ: Monocyte-macrophage
AP-MΦ: Antigen presenting-macrophages
M2-MΦ: M2-macrophages
mDC: Myeloid dendritic cells
Th1: T helper 1 cells
Th17: T helper 17 cells
Treg: Regulatory T cells
CTL-1: Cytotoxic T lymphocyte-1
CTL-2: Cytotoxic T lymphocyte-2
NKT: Natural killer T cells
PSA: Prostate-specific antigen
